# Label Free Poly(2,5-dimethoxyaniline)–Multi-Walled Carbon Nanotubes Impedimetric Immunosensor for Fumonisin B_1_ Detection

**DOI:** 10.3390/ma9040273

**Published:** 2016-04-07

**Authors:** Milua Masikini, Avril R. Williams, Christopher E. Sunday, Tesfaye T. Waryo, Ezo Nxusani, Lindsay Wilson, Sinazo Qakala, Mawethu Bilibana, Samantha Douman, Anovuyo Jonnas, Priscilla G. L. Baker, Emmanuel I. Iwuoha

**Affiliations:** 1 SensorLab, Department of Chemistry, University of the Western Cape Robert Sobukwe Road, Bellville 7535, Cape Town, South Africa; mmasikini@uwc.ac.za (M.M.); csunday@uwc.ac.za (C.E.S.); twaryo@uwc.ac.za (T.T.W.); 2861275@myuwc.ac.za (E.N.); 2724554@myuwc.ac.za (L.W.); 2346099@myuwc.ac.za (S.Q.); 2324593@myuwc.ac.za (M.B.); 2810366@myuwc.ac.za (S.D.); 3053138@myuwc.ac.za (A.J.); pbaker@uwc.ac.za (P.G.L.B.); 2 Department of Biological and Chemical Sciences, The University of the West Indies, Cave Hill Campus, Bridgetown BB11000, Barbados; avril.williams@cavehill.uwi.edu

**Keywords:** fumonisin B_1_, anti-fumonisin antibody, immunosensor, poly(2,5-dimethoxyaniline)-multi-walled carbon nanotubes, certified reference materials, electrochemical impedance spectroscopy

## Abstract

An impedimetric immunosensor for fumonisin B_1_ (FB_1_) was developed from a poly(2,5-dimethoxyaniline)-multi-walled carbon nanotube (PDMA-MWCNT) composite on the surface of glassy carbon electrode (GCE). The composite was prepared electrochemically and characterized using cyclic voltammetry. The preparation of the FB_1_ immunosensor involved the drop-coating of a bovine serum albumin mixture of the anti-fumonisin antibody (anti-Fms) onto the composite polymer-modified GCE. The electrochemical impedance spectroscopy (EIS) responses of the FB_1_ immunosensor (GCE/PDMA-MWCNT/anti-Fms) have a linear range of 7 to 49 ng·L^−1^, and the corresponding sensitivity and detection limits are 0.272 kΩ L·ng^−1^ and 3.8 pg·L^−1^, respectively. The limit of detection of the immunosensor for certified corn sample (*i.e.*, certified reference material) is 0.014 ppm FB_1_, which is in excellent agreement with the value published by the vendors and significantly more accurate than that obtained with enzyme-linked immunosorbent assay (ELISA).

## 1. Introduction

The term mycotoxin was coined in 1962 in the aftermath of an unusual veterinary crisis near London, England, during which approximately 100,000 turkey poults died [1]. Mycotoxins are secondary metabolites mainly produced by various mycelial structures of filamentous fungi growing on a wide range of agricultural commodities. The term “myco” in mycotoxin originates from the Greek word “*mykes*”, which refers to fungus [2]. Some fungi are capable of producing more than one mycotoxin, and some mycotoxins are produced by more than one fungal species [3], but in all cases, these compounds are acutely toxic to humans and animals [4]. Most mycotoxins are produced by Aspergillus, Penicillium and Fusarium, and in many cases, they are formed in the field during the growing season or during harvest, drying and storage. There are currently over 300 known mycotoxins [5], but only a relatively small number has been shown to occur in foods and feeds at levels sufficient to cause health problems.

The major classes of mycotoxins include aflatoxins, trichothecenes, fumonisins, zearalenone, ochratoxin A and the ergot alkaloids. Other mycotoxins, such as *Penicillin* acid, roquefortine, isoflumigaclavines A and B, PR toxin and cyclopiazonic acid, classified as minor [1,6], occasionally are associated with mycotoxicoses of humans and other animals or occur frequently in selected substrates, but have never been associated with human or other animal disease.

Fumonisins are produced by a number of fusarium species, especially *F. verticillioides* (previously fusarium moniliforme = *Gibberella fujikuroi*), *F. proliferatum*, *F. anthophilum*, *F. nygamai*, as well as *Alternaria alternata f.* sp. *lycopersici* [1]. Actually, over twenty-eight fumonisins have been isolated and classified into four series known as A, B, C and P. *F. verticillioides* produces several mycotoxins, the most prominent of which is called fumonisin B_1_ (FB_1_) [7]. Fumonisin B_1_ is the diester of propane-1,2,3-tricarboxylic acid (TCA) and a pentahydroxyeicosane in which the C14 and C15 hydroxy groups of the latter are esterified with the terminal carboxy group of TCA. While some toxin synthesis may occur during transportation and storage, unsurprisingly, Fusarium mycotoxins found in food are produced mainly in the field, where temperature and moisture conditions are crucial factors that favor fungal infection and toxin synthesis [7].

FB_1_, FB_2_ and FB_3_ are the principal fumonisins analyzed as natural contaminants of cereals [8,9] and can trigger serious human and animal diseases [10,11]. Exposure to fumonisin B_1_ (FB_1_) in maize causes leukoencephalomalacia (LEM) in horses and pulmonary edema in pigs. Leukoencephalomalacia has been reported in many countries, including the USA, Argentina, Brazil, Egypt, South Africa and China. FB_1_ can also harm the central nervous system, liver, pancreas, kidney and lungs in a number of animal species. For example, the presence of fumonisins in maize has been linked to the occurrence of human esophageal cancer in the Transkei, South Africa and China. The relationship between exposure to *F. moniliforme* in home-grown maize and the incidence of esophageal cancer was studied in the Transkei from 1976 to 1986 [12].

Fumonisins have been traditionally analyzed by chromatographic methods, such as thin layer chromatography (TLC), liquid chromatography (LC), liquid chromatography-mass spectrometry (LC-MS), gas chromatography-mass spectrometry (GC-MS) and high-performance liquid chromatography (HPLC), which are expensive and time-consuming methods and, despite their higher sensitivity and specificity, require appropriate instrumentation and trained personnel. As a less expensive alternative, the ELISA method has a higher detection limit, but the requirements include a stable source of antibodies and incubation time for enzyme-substrate reactions, rendering it unsuitable for use in the field [13,14]. Previously, we developed an impedimetric immunosensor for the detection of fumonisins on poly(2,5-dimethoxyaniline)-multi-walled carbon nanotubes doped with palladium telluride quantum dots onto a glassy carbon electrode surface. However it had relatively low stability [15]. Very recently, Yang *et al.* developed a practical detection method and sensitive electrochemical immunosensor for detecting fumonisin B1 (FB_1_) in corn using a single-walled carbon nanotube/chitosan composite and differential pulse voltammetry (DPV) measurements. This method, however, only allowed for the indirect identification of FB_1_ and also exhibited low stability [16]. Consequently, there is still a need for an immunosensor, for the detection and quantification of FB_1_ in food/feed, that is low cost, convenient and rapid with improved stability and higher sensitivity, such as the one presented in this study, which is based on a poly(dimethoxyaniline)-multi-walled carbon nanotube (PDMA-MWCNT) nanocomposite.

Interactions between polyaniline (PANI) and carbon nanotubes (CNT) in the composites can occur in a number of ways. It was first suggested that the attachment of aniline radicals, generated during electrochemical oxidative polymerization, onto the CNT lattice occurred at defect sites [17]. However, since then, it has been shown that the carboxylic acid sites at the acid-treated CNT are the most likely sites of interaction with the aniline monomer [18]. Electrical, thermal and mechanical properties observed in PANI-CNT composites are intermediate between pure PANI and CNT and are dependent on CNT content and the extent of its integration with PANI. Thus, the electrochemical properties of a PANI-CNT composite are enhanced compared to the two individual components. For instance, electrochemical growth together with the values of the redox and capacitive currents of the composites are several-fold greater than the pure PANI electrode [17,18]. The same behavior is also observed in composites of CNT with substituted PANI derivatives, where the extent of the increase in current depends on the nature of the substituent present on the aniline ring [17]. Such a remarkable current enhancement appears to be unique to PANI-CNT composites and has not been observed for any other conjugated polymer-CNT composite. This synergistic performance of PANI-CNT composites has been beneficial in certain applications, including fuel cells, batteries, supercapacitors, sensors and organic devices and electronics, where for example, PANI can be used as a dopant to convert a *p*-type CNT field-effect transistor (CNTFET) to an *n*-type device [19].

## 2. Results and Discussion

### 2.1. Electrochemical Synthesis of the PDMA-MWCNT Composite

**Figure 1:**
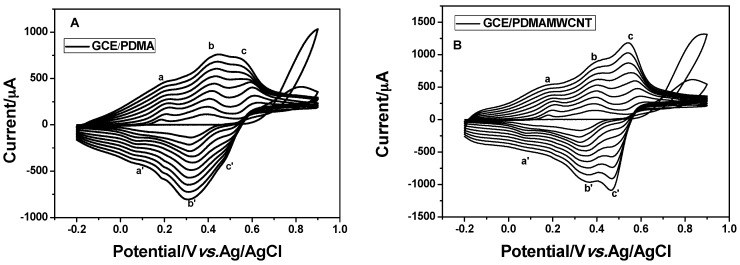
Cyclic voltammograms (CVs) for the electrochemical synthesis of (**A**) poly(dimethoxyaniline) (PDMA) and (**B**) poly(dimethoxyaniline)-multi-walled carbon nanotube (PDMA-MWCNT) films on glassy carbon electrode (GCE) in 1.0 M HCl at a scan rate of 50 mV·s^−1^.

Electrochemical polymerization of the monomer 2,5-dimethoxyaniline, on a glassy carbon electrode surface, in the absence and presence of multi-walled carbon nanotubes (MWCNT) was achieved by cycling the potential ten times between −0.2 and 0.9 V at a scan rate of 50 mV·s^−1^ (Figure 1). Successful polymerization was indicated by the formation of the green emeraldine film on the electrode surface. As the polymerization process progressed, the peak current and polymer thickness increased with the number of successive potential cycles, indicating that conductive polymeric films were formed (Figure 1). The electrodeposition of PDMA-MWCNT on the glassy carbon electrode surface proceeded via a radical cation mechanism, where two pairs of redox peaks, centered at approximately 0.18 V (a/a′) and 0.55 V (c/c′), correspond to the transition of the leucoemeraldine to emeraldine and emeraldine to pernigraniline states [20], respectively. These redox peaks indicate the presence of discrete electroactive regions in both the doped PDMA and PDMA-MWCNT films. The origin of another pair of redox peaks observed at *ca*. 0.43 V (b/b′) for both PDMA-MWCNT and PDMA is much more complex and has been attributed to many different intermediates and degradation products (the cross-linked polymer, benzoquinone, emeraldine/emeraldine radical cation, *etc.*) [21].

### 2.2. Characterization of the PDMA-MWCNT Composite

#### 2.2.1. Fourier Transform Infrared Spectroscopy

**Figure 2:**
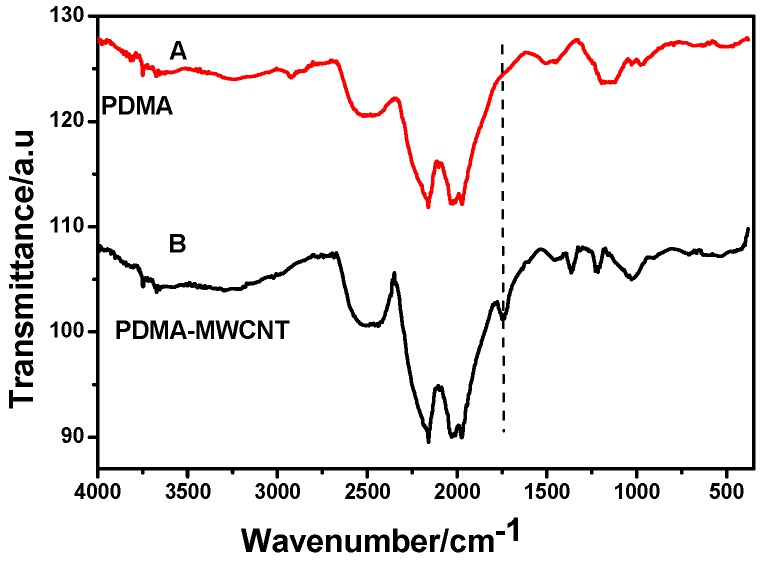
Fourier transform infrared (FTIR) spectra of (**A**) PDMA and (**B**) PDMA-MWCNT.

The structure of PDMA, before and after the introduction of MWCNT, was studied using Fourier transform infrared (FTIR) spectroscopy (Figure 2). The spectrum of PDMA (Figure 2A) exhibits the main characteristic bands in the 400 to 4000 cm^−1^ range [22,23]. A broad, weak band at ~3237 cm^−1^ is attributed to the N–H stretching mode and the peak at 1194 cm^−1^ corresponds to the quinoid rings in the polymer backbone, while the corresponding stretching vibration bands for the benzenoid rings occur at 1504 and 1447 cm^−1^. The band at ~1120 cm^−1^ is assigned to the plane bending vibration of C–H, formed during protonation, while the bands at 1028 and 982 cm^−1^ indicate the presence of the O–methoxy groups in PDMA. Lastly, a band at 800 cm^−1^ indicates the ortho-substituted benzene ring. The FTIR spectrum of PDMA-MWCNT (Figure 2B) is almost identical to that of PDMA, with the exception of some shifting of the main peaks, as a result of changes in the environment at the molecular level. The differentiating peaks at ~1732, 1364 and 1160 cm^−1^, observed only in the PDMA-MWCNT spectrum, are facets of the C=O stretch, O–H bend and C–O stretch, respectively, of the carboxylic acid group [9,24] of the MWCNT, confirming its attachment to PDMA.

#### 2.2.2. Ultraviolet-Visible Spectroscopy

**Figure 3:**
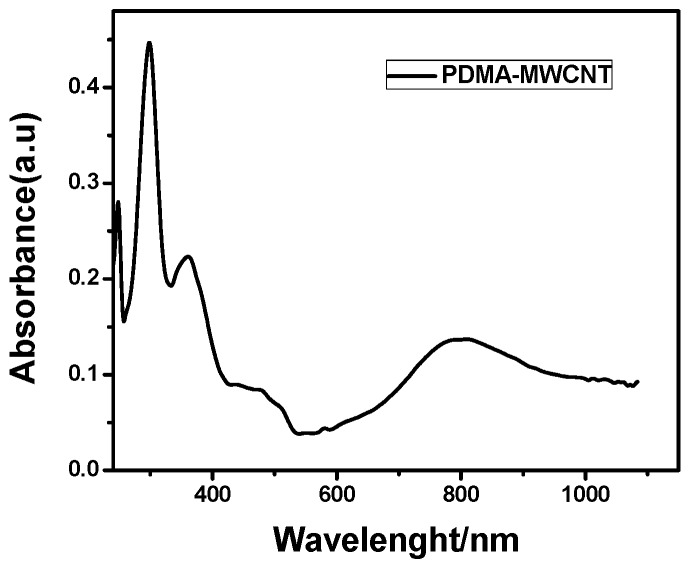
UV-visible absorption spectrum of PDMA-containing MWCNT in dimethylsulphoxide (DMSO).

To understand the electronic states of PDMA-MWCNT, ultraviolet-visible (UV-Vis) spectroscopy was the tool of choice. The UV-Vis spectrum of PDMA-MWCNT (Figure 3) showed three major absorption bands at 299, 361 and 801 nm. The peaks at 299 nm and 361 nm are attributed to the π–π* transitions of the benzenoid rings [25,26] and are assigned to the leucoemeraldine form of PDMA and the protonated form of the emeraldine salt [27], respectively. The band of the longest wavelength can be attributed to polarons (free or mobile) [27] or to polaron–π* transitions [28], which originate from the charged cationic species [29]. The broad peak at ~450 nm indicates the saline composition of the polymer.

#### 2.2.3. Electrochemical Characterization

**Figure 4:**
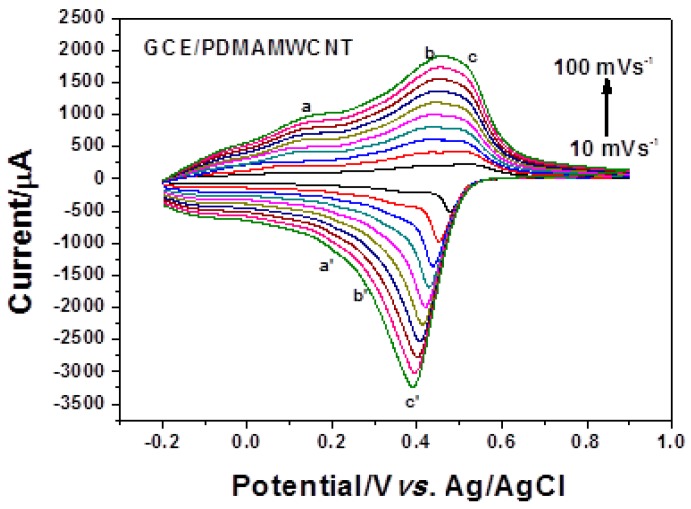
Cyclic voltammograms of the GCE/PDMA-MWCNT composite in 1 M HCl at scan rates of 10 to 100 mV·s^−1^ from the inner to the outer CVs measured in 10 mV·s^−1^ intervals.

Characterization of the electrodeposited PDMA-MWCNT composite film was spearheaded by cyclic voltammetry in 1.0 M HCl at various scan rates (10 to 100 mV·s^−1^), as shown in Figure 4.

The voltammograms exhibited three redox peaks: the first (a/a′) is representative of the leucoemeraldine radical cation/leucoemeraldine pair; the second (b/b′) is assigned to the emeraldine/emeraldine radical cation pair; and the third (c/c′) is the pernigraniline/pernigraniline radical cation couple [30]. Closer inspection of the voltammograms revealed the variation of the peak potentials and corresponding currents with scan rates confirming the successful attachment of the electroactive PDMA-MWCNT film onto the glassy carbon electrode surface.

**Figure 5:**
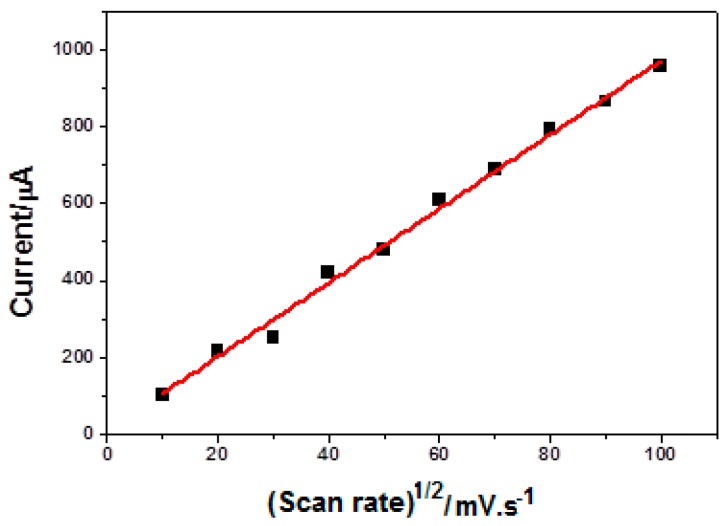
Randles–Sevčik plot for the anodic Peak a of Figure 4.

Kinetic studies of PDMA-MWCNT (using Peak a for the 10 mV·s^−1^ scan rate) enabled the estimation of a one (1) electron transfer system, which is in agreement to those reported in the literature for PDMA [31,32]. A Randles–Sevčik plot for PDMA-MWCNT (Figure 5) confirmed the presence of conducting electroactive polymers on the electrode, undergoing rapid charge transfer reactions.

The surface concentration (Г*) of the absorbed electroactive species was determined to be 1.438 × 10^−4^ mol·cm^−2^ from a plot of *I*_p_ *versus* υ in accordance with the Brown–Anson model [31,33] using Equation (1):

(1) $$I_{p}\;=\;\frac{n^{2}F^{2}\Gamma^{*}\nu A}{4RT}$$

This value was in agreement with the one obtained for glassy carbon electrode (GCE)/PDMA (1.302 × 10^−4^ mol·cm^−2^) for Peak **a**.

**Figure 6:**
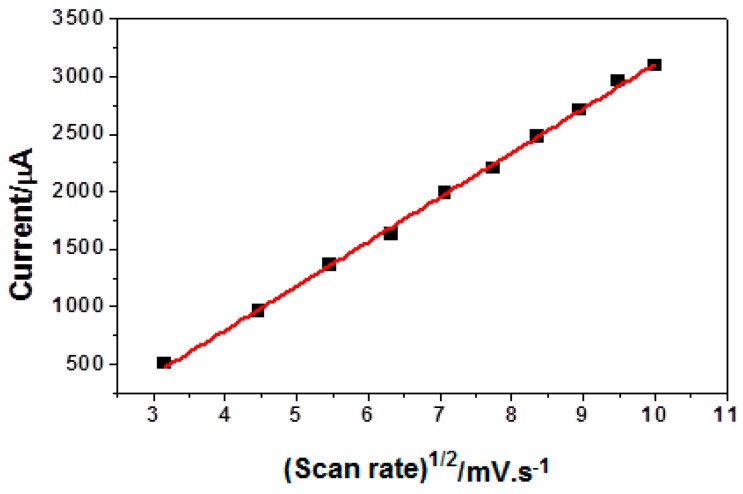
Randles–Sevčik plot for the cathodic Peak **c′** of Figure 4.

Figure 4 suggested diffusion-control of the cathodic peak current arising from the electron propagation through the polymer chain of the nanocomposite, and so, the Randles–Sevčik plot (Figure 6) and equation were used to determine the rate of charge transport coefficient (*D*) along the polymer chain. The value for GCE/PDMA-MWCNT was estimated to be 8.128 × 10^−3^ cm^2^·s^−1^, which is much higher than those reported by Iwuoha *et al.* [30], Mathebe *et al.* [31] and Klink *et al.* [32], who obtained *D* values for polyaniline-poly(styrene sulfonic acid) (8.68 × 10^−9^ cm^2^·s^−1^), polyaniline-poly(vinylsulfonic acid) (6.46 × 10^−8^ cm^2^·s^−1^) and poly(2,5-dimethoxyaniline phenanthrene sulfonic acid) (2.008 × 10^−9^ cm^2^·s^−1^), respectively, in 1 M HCl. This higher diffusion coefficient suggests increased motion of the analyte through the solution, leading to a faster electron transfer process than those reported in the literature. 

**Figure 7:**
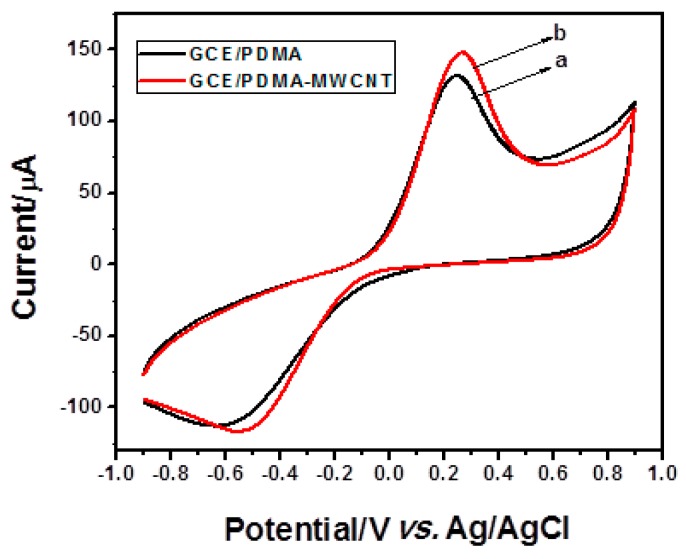
Cyclic voltammograms of (**a**) PDMA and (**b**) PDMA-MWCNT electrodes in 0.1 M phosphate buffer (PBS) at 100 mV·s^−1^.

To compare the conductivity of the two modified electrodes, cyclic voltammetry of PDMA and PDMA-MWCNT nanocomposite films was performed in 0.1 M PBS solution at a scan rate of 100 mV·s^−1^, as presented in Figure 7. The typical PDMA redox peaks (emeraldine and leucoemeraldine) of polyaniline were observed for both electrode surfaces. Peak currents of the redox couple (anodic and cathodic peak current) for PDMA-MWCNT (*I*_pa_ = 147 μA and *I*_pc_ = 118 μA) were higher than those of PDMA (*I*_pa_ = 133 μA and *I*_pc_ = 112 μA). This enhancement in current indicates fast electron transfer, which increases the charge transport in the parallel interface of PBS and the GCE/PDMA-MWCNT electrode due to the enhancement of the surface concentration of redox species in the composite.

### 2.3. Immobilization of the Antibody and Characterization of the Immunosensor

Having fully characterized the composite electrode, we then turned our attention to the immobilization of the fumonisin FB_1_ antibody and the cyclic voltammetric performance of the resulting immunosensor on standard and field samples.

**Figure 8:**
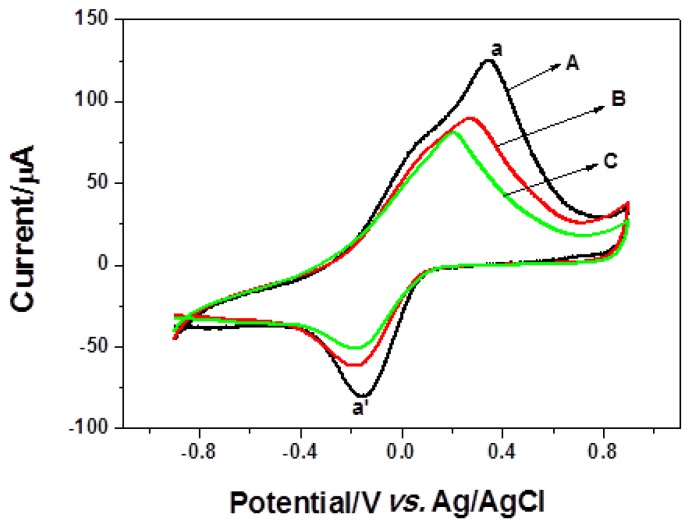
Cyclic voltammograms of (**A**) GCE/PDMA-MWCNT; (**B**) GCE/PDMA-MWCNT after immobilization of the FB_1_ antibody and (**C**) GCE/PDMA-MWCNT after immobilization and running in FB_1_ antigen in PBS at a scan rate of 50 mV·s^−1^.

Figure 8A shows the cyclic voltammetry (CV) of the GCE/PDMA-MWCNT film presenting a redox couple with the anodic peak at 341 mV (a) and the cathodic peak at −158 mV (a**′**). Similar couples, attributable to the transition of the PDMA backbone from its leucoemeraldine state (reduced form) to emeraldine state (partially oxidized form) [34], were observed for the GCE/PDMA-MWCNT after immobilization of fumonisin antibody, Fms (B), and following the antibody-antigen interaction (C). The composite electrode (A) revealed the highest current for both the anodic and cathodic peaks. The observed decrease in current on going from the composite electrode to the immunosensor and again during the testing of the immunosensor is indicative of slow electron transport and decreased charge transport in the parallel interface of the PBS solution and the GCE/PDMA-MWCNT after immobilization of the antibody. This result is not unexpected, as the antibody and antibody-antigen layers are not considered conductive materials.

**Figure 9:**
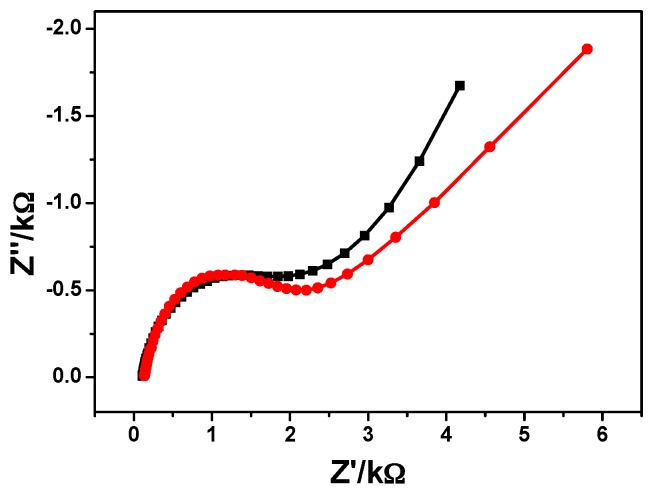
Nyquist plots for GCE/PDMA-MWCNT (black) and GCE/PDMA-MWCNT/anti-Fms (red).

The effect of anti-fumonisin antibody the sensor platform (GCE/PDMA-MWCNT) was studied by performing electrochemical impedance spectroscopy (EIS) experiments in PBS. Figure 9 shows the Nyquist diagrams of GCE/PDMA-MWCNT (black) and GCE/PDMA-MWCNT/anti-Fms (red). The sensor parameters which are summarized in Table 1, indicate no significant difference between the two electrode systems, thereby suggesting that the antibody has no significant effect on the PDMA-MWCNT layer in the absence of FB_1_ antigen.

### 2.4. Performance of the Immunosensor

**Figure 10:**
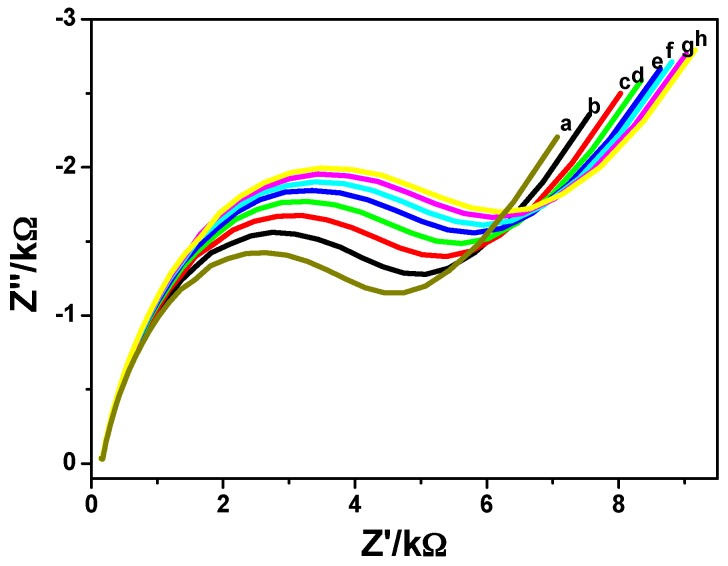
EIS responses in PBS, of the FB_1_ immunosensor (GCE/PDMA-MWCNT/anti-Fms) at the following concentrations of FB_1_: (**a**) 0 ng·L^−1^; (**b**) 7 ng·L^−1^; (**c**) 14 ng·L^−1^; (**d**) 21 ng·L^−1^; (**e**) 28 ng·L^−1^; (**f**) 35 ng·L^−1^; (**g**) 42 ng·L^−1^; and (**h**) 49 ng·L^−1^.

The responses of the GCE/PDMA-MWCNT immunosensor to FB_1_ standard were investigated using electrochemical impedance spectroscopy (EIS) at concentrations ranging from 0 to 49 ng·L^−1^ in 5 mL PBS (Figure 10). The charge transfer resistance (*R*_ct_) was seen to increase with increasing analyte concentration. The charge transfer resistance is dependent on FB_1_ concentration and is influenced by the decrease in current caused by the insulating properties of the complex formed between the fumonisin-BSA conjugate and the anti-fumonisin antibody [35] (BSA blocks non-specific binding sites on the immunosensor). Additionally, as the antigen exists as a dianion (FB_1_^2−^) at neutral pH, owing to the ionization of the carboxyl and phenol groups, binding of this charged antigen (FB_1_^2−^) to the fumonisin immunosensor increases the charge transfer resistance [36].

**Figure 11:**
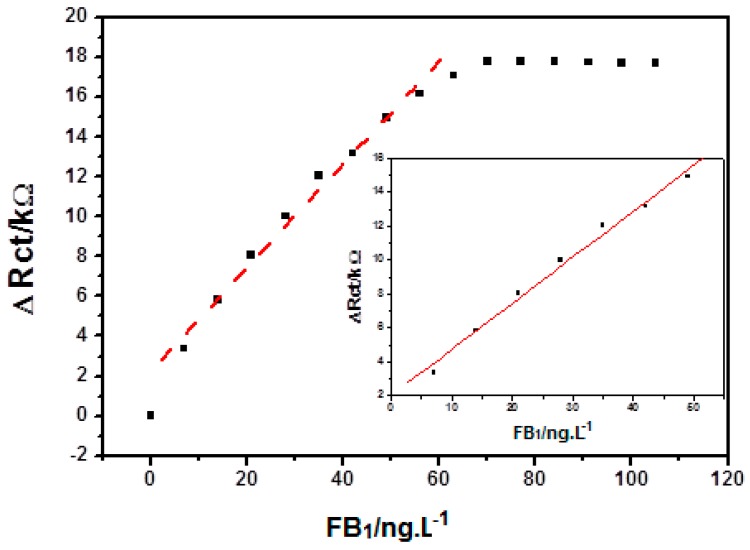
Calibration plots of the GCE/PDMA-MWCNT/anti-Fms immunosensor.

**Figure 12:**
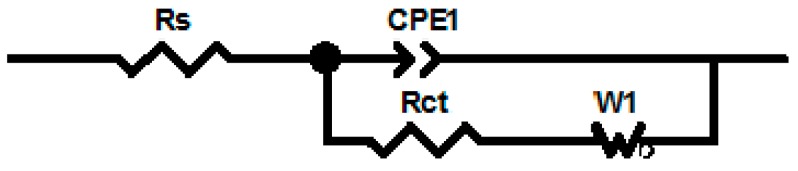
Model equivalent circuit of the GCE/PDMA-MWCNT immunosensor.

Figure 11 shows the change in the charge transfer resistance with FB_1_ concentration for GCE/PDMA-MWCNT/anti-Fms from which the sensitivity and detection limits were determined to be 0.272 kΩ L·ng^−1^ and 3.8 pg·L^−1^, respectively. The change in charge transfer resistance (∆*R*_ct_) increases exponentially with increasing concentration (0 to 105 ng·L^−1^). Above 63 ng·L^−1^, it is observed that the responses to those changes are no longer linear and plateau due to the surface saturation of adsorbed molecules of FB_1_. As such, there are no longer any available sites for FB_1_ to interact with the antibody, so no additional significant increments in the charge transfer resistance (∆*R*_ct_) are observed. Thus, the detection limit was calculated as follows:

The equivalent circuit proposed for the interpretation of the EIS measurements of the immunosensor is shown in Figure 12, where *Z*_w_ (Warburg impedance) and *R*_s_ (solution resistance of the electrolyte solution) represent the properties of the electrolyte solution and diffusion at the redox probe, and they are not affected by modifications occurring on the electrode surface. Contrastingly, *R*_ct_ (electron transfer resistance) and CPE (constant phase element) depend on the dielectric and insulating features at the electrode/electrolyte interface and are thus affected by the changes at the electrode surface. Table 2 shows the comparison of the present immunosensor to previously-reported immunosensors for fumonisin B_1_ detection, where the GCE/PDMA-MWCNT immunosensor compares favorably with those reported in the literature, and in all but the two cases (*i.e.*, 1 and 2 pg·mL^−1^) [14,16], an increase in the detection limit was realized.

### 2.5. Stability and Repeatability of the Immunosensor

The stability of the GCE/PDMA-MWCNT immunosensor was investigated by EIS in PBS containing 4 × 10^−5^ μM of FB_1_, where the temperature was held at 4 °C for five days, and daily measurements were obtained. The results showed that the immunosensor retained 81% of its activity, indicating that the platform was stable and that the antibodies remained firmly attached to the surface of the electrode. The repeatability of the immunosensor was evaluated by five successive measurements in the presence of 5 × 10^−5^ μM of FB_1_, where the relative standard deviation with EIS was 1.71% for GCE/PDMA-MWCNT, which is well within the experimental error.

### 2.6. Analysis of Certified Corn Reference Material

Finally, we applied the GCE/PDMA-MWCNT/anti-Fms immunosensor in the detection of fumonisins extracted from certified corn reference material, where the concentrations of fumonisin obtained were in agreement with those reported by the vendor, but at least two orders of magnitude less than those obtained using ELISA (Table 3).

## 3. Experimental Section

### 3.1. Chemicals and Reagents

The 2,5-dimethoxyaniline (98%) was purchased from Aldrich and Fluka. Fumonisin B_1_ (FB_1_) from fusarium moniliforme, received from Sigma-Aldrich, was dissolved in methanol at 1 mg/mL and stored as aliquots in tightly-sealed vials at −20 °C. A monoclonal antibody of fumonisin from mouse was supplied by Antibodies-online GmbH, Aachen, Germany (Catalogue Number ABIN346857). The antibody was lyophilized from 200 µg of protein A purified antibodies and was quoted as specific to fumonisin; immunogen: BSA-fumonisin; isotype: IgG1/Lambda; cross-reactivity not yet tested. The antibody was also divided into aliquots and stored at −20 °C until use. Certified corn reference material was purchased from Trilogy^®^ (Washington, D.C., USA), and the ELISA kit (Veratox for fumonisin: range of 1 to 6 ppm) was purchased from Neogen corporation (St. Joseph, MI, USA). Home-grown carbon nanotubes (CNTs; diameter of 40 to 200 nm and lengths up to 20 μm) were synthesized according to the method of Ndungu *et al.* [40]. Hydrochloric acid (HCl), bovine serum albumin (BSA) and absolute ethanol were purchased from Sigma-Aldrich (Johannesburg, South Africa). Basic salts, including NaH_2_PO_4_, Na_2_HPO_4_ and KCl, used in the preparation of 0.1 M phosphate buffer saline containing 0.1 M KCl at pH 7.4 (PBS), were also obtained from Sigma-Aldrich. Phosphate-buffered saline containing KCl and NaCl (10 × PBS), but diluted to 1 × PBS to realize pH 7.4, with a concentration of 0.1 M (PBS), was obtained from Antibodies-online. All electrochemical measurements for fumonisin B_1_ (FB_1_) were carried out in 0.1 M phosphate-buffered saline. All other chemicals were of analytical grade, and deionized water (18.2 MΩ cm) purified by a Milli-Q™ system (Millipore SA (PTY) Limited, Johannesburg, South Africa) was used as the reagent water for aqueous solution preparation, while analytical-grade argon (Afrox, Johannesburg, South Africa) was used for degassing.

### 3.2. Instrumentation

Electrochemical experiments were carried out with an Epsilon EC Epsilon Eclipse potentiostat (BioAnalytical Systems Incorporated (BASi), West Lafayette, IN, USA). The CV and EIS measurements were recorded on a Zahner IM6ex electrochemical workstation (Zahner-Elektrik GmbH, Kronach, Germany) using electrodes from BASi (West Lafayette) in a three-electrode electrochemical cell. Impedimetric data and voltammograms for all electrochemical experiments were recorded with computers interfaced to the Zahner workstation and the EC Epsilon Eclipse potentiostat, respectively. A glassy carbon electrode (GCE) of an area of 0.071 cm^2^ and 3 mm in diameter was used as the working electrode. A platinum wire from Sigma Aldrich and Ag/AgCl electrodes from BASi were used as auxiliary and reference electrodes, respectively. Alumina powders and micro-cloth pads were obtained from Buehler (Lake Bluff, IL, USA) and were used for the polishing of the GCE.

### 3.3. Electrochemical Synthesis of Poly(2,5-dimethoxyaniline) Multi-Walled Carbon Nanotube Composite-Modified Glassy Carbon Electrode

Before the electrosynthesis of PDMA-MWCNT on the working GCE, the surface of the electrode was preconditioned using the following procedure: the GCE was first polished with 0.3- and 0.05-mm alumina slurries and then rinsed with distilled water. The electropolymerization mixture was prepared by mixing 0.1 M 2,5-dimethoxyaniline (DMA) in 1.0 M HCl (5 mL) and MWCNT (100 μL) (if necessary), and the mixture was degassed with argon for 10 min before electropolymerization. Doped or undoped 2,5-dimethoxyaniline was polymerized on the surface of the GCE by scanning the working electrode potential repeatedly between −200 and +900 mV for 10 cycles at a scan rate of 50 mV/s. The PDMA-MWCNT and PDMA-modified GCE are denoted as GCE/PDMA-MWCNT and GCE/PDMA, respectively.

### 3.4. Fabrication of the Immunosensor

The optimal concentration of the anti-fumonisin antibody required for the immunosensor was determined by preparing sensors with different concentrations of anti-fumonisin antibody (0.005, 0.01, 0.02, 0.1 and 0.2 μg·μL^−1^) and comparing their electrochemical responses. 20 μL of the anti-fumonisin antibody sample was drop-coated on GCE/PT-PDMA-MWCNT, and cyclic voltammetry was performed in PBS (pH 7.4) at a potential range of −900 to +900 mV and a scan rate of 20 mV·s^−1^ (figure not shown). It was found that the highest redox peak currents were obtained with 0.1 μg·μL^−1^. Thus 0.1 μg·μL^−1^ anti-fumonisin antibody was used for the preparation of the immunosensor reported in this study.

**Scheme 1:**
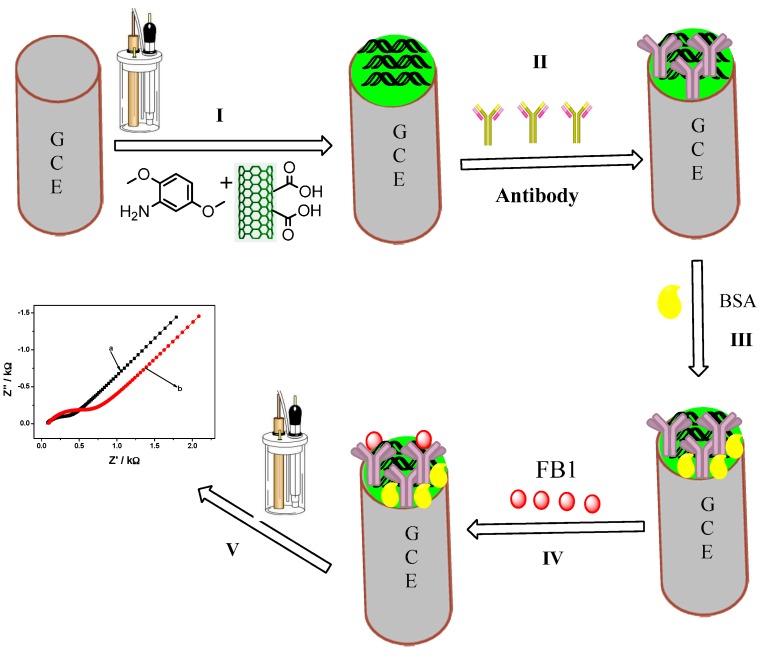
Schematic representation of the synthesis of the immunosensor. (**I**) Electrochemical synthesis of PDMA-MWCNT; (**II**) attachment of antibody by drop-coating onto the electrode surface and drying for 24 h; (**III**) addition of BSA to block non-specific binding sites; (**IV**) addition of FB_1_; (**V**) electrochemical measurements (EIS).

After the electrochemical synthesis of PDMA-MWCNT, 20 μL of 0.1 μL·μg^−1^ anti-fumonisin antibody (anti-Fms) were drop-coated on GCE/PDMA-MWCNT and allowed to dry at 4 °C for 24 h. After the immobilization of the antibody, the electrodes were rinsed with PBS in order to remove physically-unbound antibody, and then, 40 μL of BSA (0.04 mg·μL^−1^) were applied for 2 h at room temperature to block nonspecific binding sites. The immobilized electrode, denoted as GCE/PDMA-MWCNT/anti-Fms, was rinsed again with PBS, before any electrochemical measurements were performed. The entire process is represented in Scheme 1.

### 3.5. Extraction of Fumonisins from Certified Corn Reference Materials

Extraction was conducted by following the procedure described by the Veratox Elisa Kit. Preparation of a sample of the certified ground corn reference material involved the addition of 5 g of the sample to 25 mL of a 7:3 solution of methanol (HPLC grade) and deionized water in 50-mL centrifuge screw cap vials. The resulting mixture was shaken vigorously for 3 min and centrifuged for 15 min at 4500 rpm, after which the supernatant was extracted through Whatman filter paper. The filtrate was then collected for analysis without further preparation. Aliquots (10 μL) of this filtrate were used to successively spike the 5 mL PBS solution in the electrochemical cell for EIS measurements of Fms.

## 4. Conclusions

In this study, an impedimetric immunosensor, based on an electrochemically-synthesized polymer-multi-walled carbon nanotube platform, for the detection of fumonisin B_1_ was developed. The modification of the electrode surface and the interaction between the fumonisin antibodies and fumonisins was studied by electrochemical impedance spectroscopy. The measured electron transfer resistance was used to determine the amount of FB_1_ bound to the immunosensor. It was observed that the antibody layer and antibody-antigen interaction were not conductive, hence inhibiting the electron transfer process of the developed immunosensor. The immunosensor exhibited a limit of detection of 3.8 pg·L^−1^ with good sensitivity of 0.27215 kΩ·L·ng^−1^ for FB_1_ and good stability and repeatability within experimental error, all of which agree well for the use of the immunosensor in the trace detection of fumonisins. The response of the GCE/PDMA-MWCNT/anti-Fms immunosensor to certified corn reference material was an improvement on what is obtained with ELISA and it is in good agreement with the values advertised by the vendors of the reference material.

## Figures and Tables

**Table 1:** Electrochemical impedance spectroscopy (EIS) parameters for GCE/PDMA-MWCNT and GCE/PDMA-MWCNT/anti-Fms.

ELEMENT	GCE/PDMA-MWCNT	GCE/PDMA-MWCNT/Anti-Fms
Solution resistance (ohm)	128.6	132.1
Charge transfer resistance (ohm)	1129	1214
Constant phase element (F)	4.228 × 10^−6^	3.6204 × 10^−6^

**Table 2:** Characteristics of the GCE/PDMA-MWCNT immunosensor along with those reported in the literature for fumonisin B_1_ detection.

Immunosensor	Range of Linearity (ng·L^−1^)	Detection Limit (pg·L^−1^)
GCE/PDMA-MWCNT/anti-Fms (this work)	7 to 49	3.8
Gold screen-printed electrode (SPGE) [37]	1 to 1 × 10^6^	5 × 10^6^
GCE/Au nanoparticles (AuNPs) and graphene/thionine nanocomposite (GSTH) [14]	1 to 1 × 10^6^	1 × 10^−3^
Magnetoimmunosensor involving magnetic beads and disposable carbon screen-printed electrode (CSPE) [38]	0 to 5 × 10^3^	0.33 × 10^6^
Modified magnetic beads on screen-printed electrode [39]	0 to 54 × 10^3^	0.58 ± 0.05 × 10^6^
Single-walled carbon nanotubes (SWCNT)/chitosan-modified GCE [16]	1 to 1 × 10^6^	2 × 10^−3^

**Table 3:** Fumonisin content of certified corn reference materials.

Commodity	Mycotoxins	Immunosensor (ppm)	Vendor (ppm)	ELISA (ppm)
Corn	FB_1_ + FB_2_ + FB_3_	0.014 ± 0.00057	-	1.18
Corn	FB_1_	0.014 ± 0.00153	0.01	0.88
